# Diagnosis and Management of Upper Lip Sialolithiasis Identified via Conventional Dental Radiography: A Case Report

**DOI:** 10.1155/crid/2591550

**Published:** 2026-09-01

**Authors:** Makoto Matsubara, Taku Kawai, Shiro Tanaka, Motohiko Nagayama

**Affiliations:** ^1^ Department of Oral and Maxillofacial Surgery, Matsunami General Hospital, Gifu, Japan, matsunami-hsp.or.jp; ^2^ Department of Oral and Maxillofacial Surgery, Shimada General Medical Center, Shizuoka, Japan; ^3^ Department of Oral Pathology, Division of Oral Pathogenesis and Disease Control, Asahi University School of Dentistry, Gifu, Japan, asahi-u.ac.jp

**Keywords:** case report, dental radiography, minor salivary gland, sialolithiasis, upper lip

## Abstract

Sialolithiasis of the minor salivary glands is a rare clinical entity that is seldom detected through preoperative imaging. Due to its nonspecific clinical presentation, it is frequently misdiagnosed as mucocele, fibroma, or minor salivary gland tumor; consequently, most cases are identified only through postoperative histopathological examination. We report a rare case of sialolithiasis in a minor salivary gland of the upper lip in a 73‐year‐old male patient. Despite the lesion’s small size, a history of suppuration prompted radiographic investigation. A standard dental radiograph identified a 0.5‐mm radio‐opaque entity, enabling an accurate preoperative diagnosis. This case underscores that dental radiography remains a simple, cost‐effective, and highly efficient screening tool for differentiating sialolithiasis from other soft‐tissue lesions in the lip.

## 1. Introduction

Sialolithiasis is a disease characterized by the formation of calcareous concretions, or sialoliths, within the salivary gland parenchyma or ductal system [1]. It predominantly affects the major salivary glands, with the submandibular gland accounting for 80%–90% of cases and the parotid gland for 6%–20%; in contrast, minor salivary gland sialolithiasis is extremely rare, accounting for less than 1%–2% of all cases [2–4]. Unlike major salivary gland sialolithiasis, which typically develops in middle‐aged adults, minor salivary gland sialolithiasis tends to affect older populations, with a peak incidence in the 5th–6th decades of life and no clear sex predilection [3, 5]. In minor salivary glands, patients typically present with an asymptomatic, firm, submucosal nodule, and rarely exhibit “salivary colic” (i.e., pain and swelling associated with meals) due to the absence of a long duct system [4]. Consequently, it is frequently misdiagnosed as a mucocele, fibroma, or benign minor salivary gland tumor, with most cases identified only through postoperative histopathological examination [6, 7]. Furthermore, although stones in major salivary glands often reach several millimeters or centimeters in size, minor salivary gland calculi are significantly smaller, usually measuring only a few millimeters or less [5, 6]. Because of their diminutive size, these stones are often overlooked by advanced imaging modalities such as computed tomography (CT) or magnetic resonance imaging (MRI) [7]. Here, we present a case of upper lip minor salivary gland sialolithiasis that was successfully diagnosed preoperatively using conventional dental radiography and subsequently confirmed histopathologically.

## 2. Case Presentation

**Figure 1:**
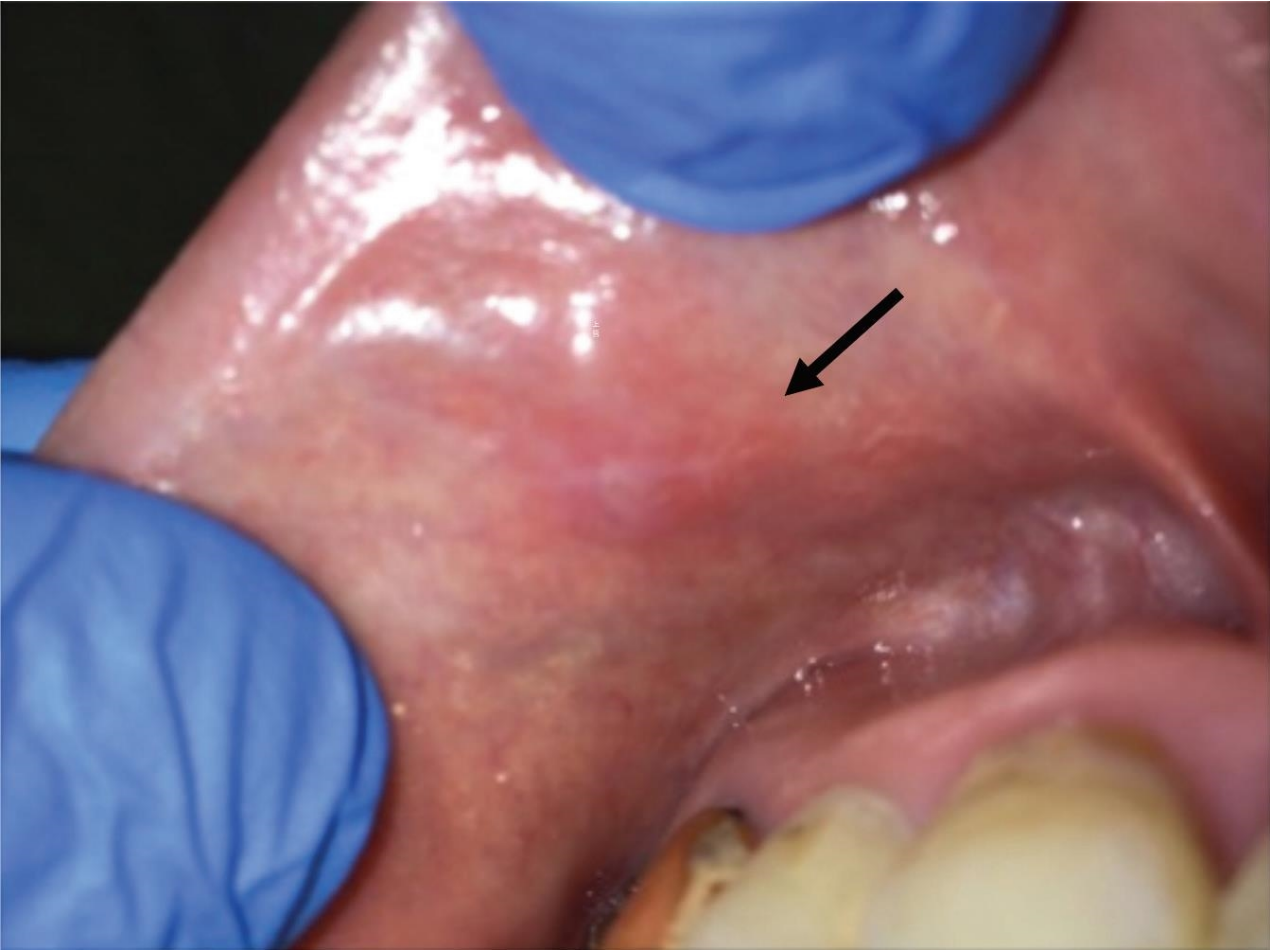
Intraoral findings at the initial administration. A nodule on the mucosal surface of the right upper lip (arrow).

A 73‐year‐old male patient presented to our department with a 6‐month history of a persistent mass in the right upper lip. The patient initially sought treatment at a local dental clinic after the mass became suppurated. Although a course of oral antibiotic therapy (amoxicillin, 250 mg three times daily for 3 days) was administered and the acute symptoms subsided, the nodule remained. Upon clinical examination, the patient showed no systemic signs of infection, such as fever or malaise. Intraoral inspection revealed a slightly erythematous, elastic‐hard nodule approximately 5 mm in diameter, located on the mucosal surface of the right upper lip (Figure 1). There was no tenderness on palpation, and no active suppuration or discharge was observed at the time of consultation.

**Figure 2:**
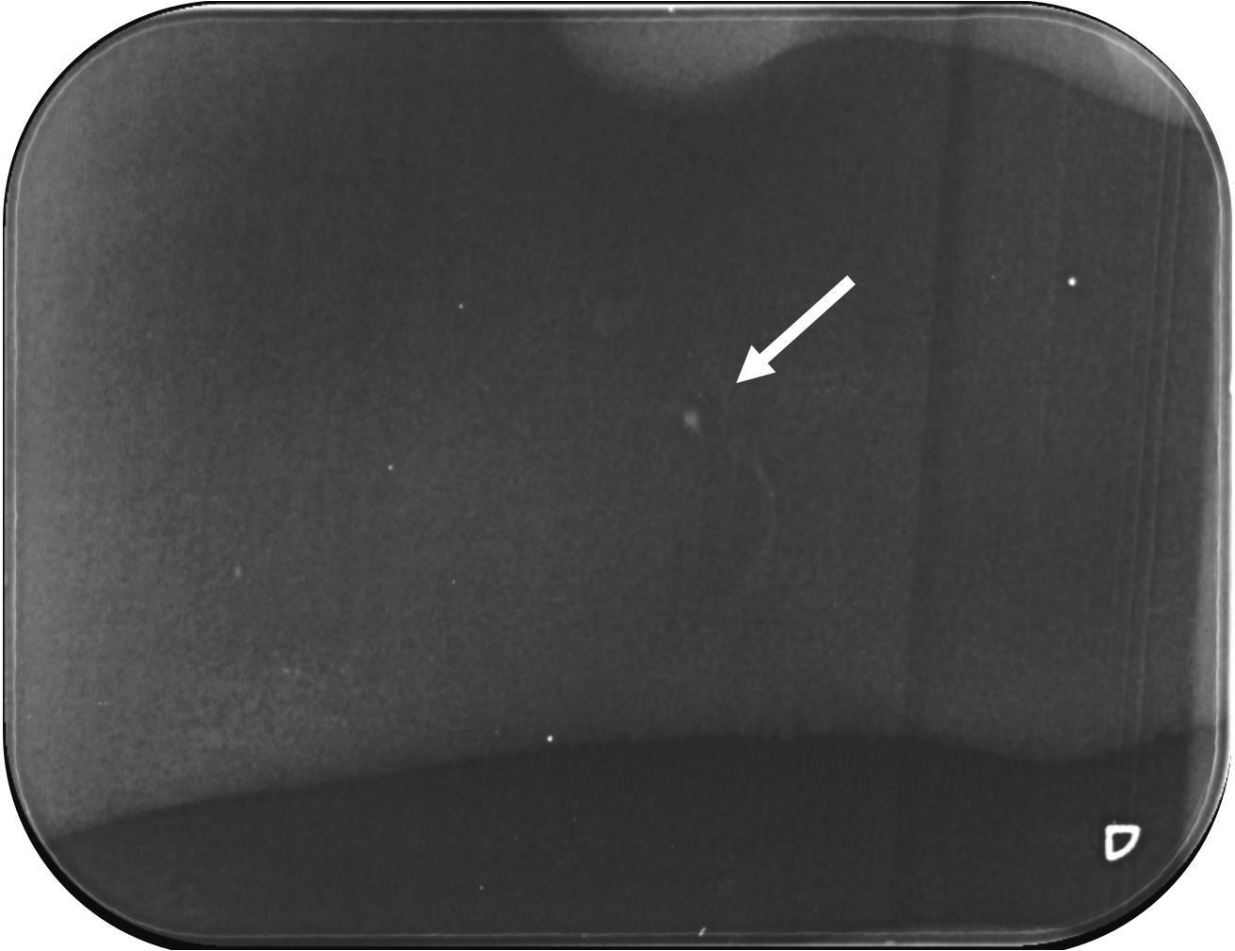
Radiographic findings at the initial visit. Dental radiograph showing a radio‐opaque entity within the soft tissue of the upper lip. An irregularly shaped radio‐opaque object is observed (arrow).

Given the history of inflammatory discharge, a dental radiograph was obtained to evaluate calcified structures within the soft tissues. The radiograph revealed a distinct 0.5‐mm radio‐opaque entity situated in the soft tissue of the upper lip, inferior to the right alar cartilage (Figure 2). Based on the radiographic evidence and clinical history, a preoperative diagnosis of sialolithiasis of the minor salivary gland was established.

**Figure 3:**
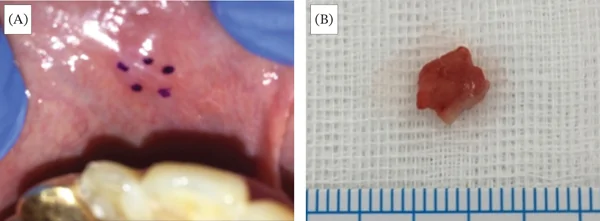
(A) Intraoperative photograph and (B) the excised specimen.

**Figure 4:**
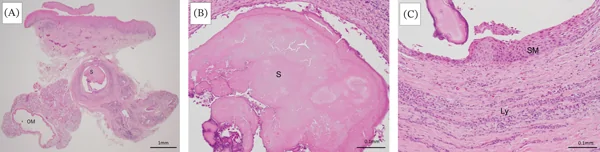
Histopathological findings. (A) Low‐magnification view showing the association with the minor salivary gland and the dilated salivary duct and (B,C) high‐magnification views. An irregularly shaped sialolith (S) within the duct. Squamous metaplasia (SM) of the ductal epithelium. Scanty lympho‐plasmocytic inflammatory infiltration (Ly) in the surrounding granulation tissue.

Under local anesthesia, the lesion was surgically excised (Figure 3A). The procedure involved a mucosal incision directly over the nodule, followed by the complete removal of the stone along with the associated minor salivary gland tissue and overlying erythematous mucosa (Figure 3B). This approach was chosen to ensure complete removal and to facilitate histopathological confirmation. Histopathological examination revealed minor salivary glands and irregularly dilated ducts beneath the labial mucosa. Some dilated ducts contained mucus with eosinophilic oncocytic metaplasia, whereas others contained irregularly shaped sialoliths showing pale eosinophilic staining. Squamous metaplasia was also observed in segments of the dilated ducts. The periductal area surrounding the sialoliths was characterized by chronic inflammatory cell infiltration, primarily lymphocytes and plasma cells, with collagen fibers forming granulation tissue, consistent with chronic sialadenitis (Figure 4). The postoperative course was uneventful, and the surgical site showed excellent healing at the 2‐week follow‐up examination. No signs of recurrence have been observed.

## 3. Discussion

Sialolithiasis of the minor salivary glands is a relatively rare condition, with the buccal mucosa and upper lip being the most common sites [3, 8]. This anatomical distribution is attributed to the higher density of minor glands in these regions and their increased susceptibility to local mechanical trauma, which can induce ductal injury, stenosis, and subsequent calculus formation [5]. Although minor salivary gland sialoliths often present as small, asymptomatic nodules, they can occasionally trigger severe local suppuration or extensive inflammatory responses, such as facial cellulitis, exhibiting diverse clinical presentations [9].

In the present case, the patient was 73 years old, aligning with the reported peak incidence of minor salivary gland sialolithiasis in the 5th–7th decades of life [2, 6]. Age is a recognized contributing factor in sialolith formation due to progressive age‐related changes in the salivary gland parenchyma, including acinar atrophy, periductal fibrosis, and ductal metaplasia [10]. These degenerative alterations, combined with reduced salivary flow, promote stasis of secretions and organic matrix precipitation, creating a favorable microenvironment for stone crystallization. This mechanism is strongly supported by our histopathological findings, which demonstrated oncocytic and squamous metaplasia of the ductal epithelium alongside chronic inflammatory cell infiltration.

Furthermore, alhtough sialolithiasis in the major salivary glands is well known to cause secondary bacterial sialadenitis due to ductal obstruction, a similar clinicopathological relationship exists in the minor salivary glands. In our patient, the clinical history of acute suppuration that initially prompted consultation at a primary dental clinic—and temporarily subsided following oral amoxicillin therapy—reflects an episode of acute secondary sialadenitis superimposed on ductal blockage. The subsequent histopathological identification of periductal granulation tissue and plasma cell infiltration confirmed the presence of underlying chronic sialadenitis resulting from long‐standing ductal irritation.

Preoperative diagnostic imaging of small sialoliths remains challenging. Although ultrasonography is a noninvasive, sensitive tool for detecting soft‐tissue lesions in the lip, its diagnostic accuracy depends heavily on operator expertise and equipment accessibility [4, 11]. Advanced modalities such as CT and MRI often fail to depict such minute calcifications due to limited spatial resolution or metallic/motion artifacts [12]. In contrast, conventional dental radiography (periapical view) proved to be a highly effective, low‐cost, and readily available screening method in this case. By placing the film intraorally and optimizing the projection angle to project the upper lip clear of the maxilla and teeth, even a 0.5‐mm sialolith was distinctly identified.

Nevertheless, clinicians must exercise caution when interpreting radiopaque or nodular lesions of the lip. In addition to phleboliths, calcified lymph nodes, and salivary gland neoplasms, radiopaque artifacts or foreign bodies—such as displaced dental calculus, metallic fragments, or fractured tooth particles secondary to prior trauma—can occasionally mimic soft‐tissue calcifications on dental radiographs [13]. Notably, minor salivary gland malignancies, such as adenoid cystic carcinoma, can clinically mimic sialolithiasis or rarely coexist with salivary calculi [12]. Therefore, a thorough intraoral examination to confirm the absence of dental calculus or traumatic debris in the lip is essential prior to definitive surgical intervention.

Regarding surgical intervention, although surgical lasers are sometimes discussed as alternative tools for oral soft‐tissue procedures, conventional scalpel excision remains the preferred standard of care for minor salivary gland sialolithiasis for two critical reasons. First, effective treatment of minor salivary gland lesions requires not only the removal of the sialolith itself, but also the total extirpation of the associated minor salivary gland acini to prevent disease recurrence, a principle well‐established in the management of minor gland pathology [14]. Second, because preoperative imaging cannot definitively rule out minor salivary gland malignancies, laser‐induced thermal vaporization risks damaging tissue margins and compromising precise histopathological evaluation. Complete surgical excision with a scalpel ensures intact specimen retrieval for definitive pathological verification.

In this patient, malignancy was definitively excluded based on clinical examination showing a well‐circumscribed, noninvasive nodule without ulceration, combined with comprehensive histopathological evaluation of the excised specimen, which demonstrated no cellular atypia, mitotic figures, or invasive growth patterns. Therefore, although targeted dental radiography provides an indispensable lead for preoperative identification, complete surgical excision with a scalpel and subsequent histopathological verification remain the gold standard for both diagnosis and management.

## 4. Conclusion

Minor salivary gland sialolithiasis of the upper lip is a rare entity that poses diagnostic challenges due to its small size and potential to mimic benign or malignant lesions. Complete surgical excision with comprehensive histopathological evaluation remains the gold standard for definitive diagnosis and treatment, particularly to rule out malignancy. However, as demonstrated in this case, a carefully positioned conventional dental radiograph provides a highly accessible, low‐cost, and effective preoperative screening tool for identifying even submillimeter calcifications in the labial soft tissues.

## Funding

No funding was received for this manuscript.

## Ethics Statement

Ethical approval was not required for this single anonymized case report. According to the regulations of our institution, ethics approval is not required for retrospective case reports involving a single patient when all patient identifiers have been removed, and the study does not alter or interfere with routine clinical practice. Written informed consent was obtained from the patient for publication of this case report and any accompanying images. A copy of the signed consent form is retained by the corresponding authors and is available upon request. This report was prepared in accordance with the Declaration of Helsinki.

## Conflicts of Interest

The authors declare no conflicts of interest.

## Data Availability

The data that support the findings of this study are available on request from the corresponding author. The data are not publicly available due to privacy or ethical restrictions.
